# Evaluation of Varicella Immunity during Pregnancy in Apulia Region, Southern Italy

**DOI:** 10.3390/vaccines8020214

**Published:** 2020-05-10

**Authors:** Claudia M. Trombetta, Emanuele Montomoli, Simonetta Viviani, Rosa Coluccio, Serena Marchi

**Affiliations:** 1 Department of Molecular and Developmental Medicine, University of Siena, Via Aldo Moro 2, 53100 Siena, Italy; emanuele.montomoli@unisi.it (E.M.); simoviviani56@gmail.com (S.V.); rosa.coluccio@gmail.com (R.C.); serena.marchi2@unisi.it (S.M.); 2 VisMederi srl, Strada del Petriccio e Belriguardo 35, 53100 Siena, Italy

**Keywords:** varicella-zoster virus, pregnancy, Apulia region, Italy

## Abstract

Varicella is a highly contagious, infectious disease caused by the varicella-zoster virus. Those at higher risk of severe complications are immunocompromised individuals, adults, non-immune pregnant women, and newborns. According to the gestational time, when varicella-zoster virus infection is acquired during pregnancy, serious complications can potentially occur for both the woman and the fetus. The present study was conducted to assess the profile of varicella susceptibility in pregnant women in Apulia, a large region in Southern Italy, from 2016 to 2019. The data showed that pregnant women between the age of 15–24 and 40–49 years, the youngest and the oldest, respectively, are the most protected against varicella-zoster virus infection, exceeding the prevalence rate of 90%. Conversely, pregnant women between the age of 25 and 34 years seem to be the most vulnerable and the most at risk for acquiring varicella-zoster virus infection during pregnancy. Analysis of the immunity status against varicella should be introduced as a screening test before pregnancy, together with a strategic vaccination campaign targeting non-immune women of childbearing age, in order to reduce the risk of congenital and perinatal varicella.

## 1. Introduction

Varicella is a highly contagious, infectious disease caused by the varicella-zoster virus (VZV). In temperate climates, approximately 90% of infections occur within the first 15 years of life. Typically, disease severity increases with age, with a 25-fold probability of serious clinical outcomes in adults compared to children. Those at a higher risk of severe complications are immunocompromised individuals, adults, non-immune pregnant women, and newborns. Primary infection usually confers lifelong immunity, however a reactivation of the virus may lead to herpes-zoster (HZ) infection, usually occurring in immunocompromised or elderly individuals [1,2,3,4,5].

According to the gestational time, when VZV infection is acquired during pregnancy, serious complications can potentially occur for both the woman and the fetus. Maternal infection during the first trimester of pregnancy can lead to spontaneous abortion, or, if acquired during the initial two trimesters, to congenital varicella syndrome (CVS), a condition characterized by several fetal abnormalities such as lesions of the skin, skeletal deformities, fetal malformation, growth restriction and neurological defects [5,6,7]. Among infected fetuses, 12% show signs of CVS and 30% die during the first few months of life [7,8]. The incidence of congenital anomalies after maternal infection during the first 20 weeks of pregnancy is estimated to be around 2% [9]. On the other hand, infection during the last trimester of pregnancy may increase the risk of maternal pneumonia [2,5]. In the case of ultrasound evidence of fetal compromise, an amniocentesis can be taken into account, according to the gestational age. For the prenatal detection of VZV infection, amniocentesis has a strong negative predictive value but a low positive predictive value. Therefore, for women who get the infection during pregnancy, a frequent ultrasound assessments are suggested, preserving amniocentesis for viral DNA investigation just for those reporting a fetal malformation, in order to confirm the diagnosis and exclude further possible reasons responsible for the abnormality. The absence of ultrasound abnormalities does not exclude a fetal infection; indeed, some lesions are not detectable through the scan. During the second part of the pregnancy, prenatal magnetic resonance imaging can be useful to diagnose or better detail damage to the central nervous system [10]. Neonatal varicella occurs when a maternal infection is acquired during the last three weeks of pregnancy and can result in very serious outcomes if acquired from 5 days before to 2 days after delivery. In the latter case, the mortality of newborns is high (30%) due to exposure to a high viral load in the absence of maternal protective immunity. Notably, the newborns could develop HZ during the first year of life regardless of the maternal timing of acquisition of VZV infection [1,7].

Until 2017, no national recommendations against varicella were in place in Italy, when it was included in the National Vaccine Prevention Plan (PNPV) as a mandatory vaccination for the 2017 birth cohort, and highly recommended for women of childbearing age [11]. Since 2003, however, eight Italian regions, representing almost 40% of the Italian population [12], have introduced the universal routine vaccination (URV) against varicella for children. Since 2006, the Apulia region, a large region in the south of Italy, started offering the varicella vaccination as a URV as a single dose to children between 12–24 months of age. Since 2009, a second dose at 5–6 years of age was added and varicella vaccination was offered as a catch-up campaign to all susceptible adolescents [13]. This approach significantly increased varicella vaccination coverage in the Apulia region from 49% in the 2006 birth cohort to 91.1% in the 2010 birth cohort, with a significant reduction in disease incidence and hospitalization [14].

Similarly to measles [15], the identification of women at risk of developing varicella during pregnancy is not recommended in Italy. However, we believe that serological studies to evaluate the prevalence of varicella antibodies in pregnant women can answer the question of whether a varicella immunity screening program is needed in this high-risk group.

The present study was conducted to assess the profile of varicella susceptibility in pregnant women in Apulia, a large region in Southern Italy, from 2016 to 2019.

## 2. Materials and Methods 

### 2.1. Study Population

Serum samples of pregnant women were collected from February 2016 to August 2019 in the province of Bari, which has the highest population density in Apulia. Serum samples were anonymously collected in compliance with Italian ethics law and stored at the sera bank of the laboratory of Molecular Epidemiology of the University of Siena, Italy. For each serum sample, the available information was age, sex, state of pregnancy, place and year of sampling. For samples collected from August 2017, information on gestational period was also available.

Assuming an overall VZV IgG prevalence of 89% [16], a precision of the estimate of 3% and a confidence interval of 95%, a sample size of 405 serum samples was required.

A total of 507 samples were selected from the sera bank and stratified by age groups (15–24, 25–29, 30–34, 35–39, 40–49 years of age), as shown in Table 1. The mean age was 33.2 ± 5.1 years.

Out of 507 samples, 287 had information on gestational period, reported as weeks of pregnancy. These samples were further stratified by trimester of pregnancy (Table 2), according to the National Institutes of Health’s (NIH) definition: first trimester from week one to week 12, second trimester from week 13 to week 28, third trimester from week 29 to week 40 [17].

### 2.2. Serological Assay

Specific VZV IgG and IgM were detected by commercial ELISA kits (Enzywell Varicella IgG and Enzywell Varicella IgM; DIESSE, Siena, Italy). Tests were performed following the manufacturer’s instructions.

Samples were considered positive for IgM and IgG when the ratio between the optical density (OD) of the sample and that of the cut-off was > 1.2 and negative when the ratio between the OD of the sample and that of the cut-off was < 0.8. In accordance with the manufacturer’s instructions, samples with a borderline result (± 20% of the cut-off) were retested.

### 2.3. Statistical Analysis

IgM and IgG antibody prevalence rates were calculated along with their corresponding 95% confidence intervals (95% CI). Chi-square tests for trend were used for statistical analysis using GraphPad Prism 6 software; statistical significance was set at *p* < 0.05, two-tailed.

## 3. Results

Out of 507 samples tested, 20 were borderline for IgG and one for IgM. After retesting, one sample for IgG and one sample for IgM still showed borderline results. 

**Figure 1:**
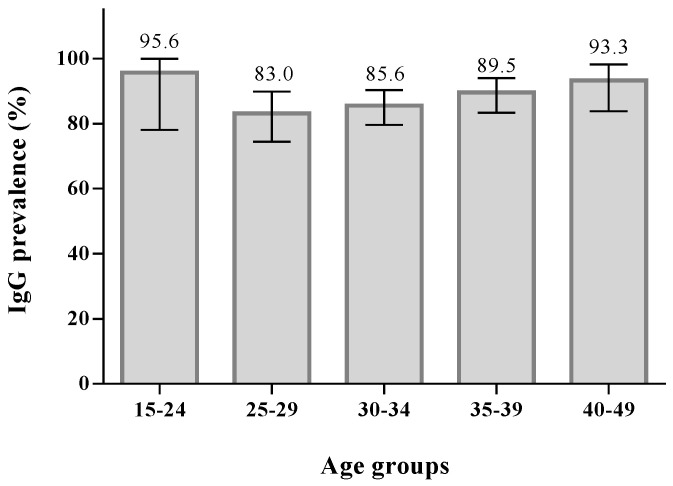
Prevalence of anti-VZV IgG antibodies in pregnant women by age groups: 95.6% (95% CI: 78.0–99.9) in 15–24 age group, 83.0% (95% CI: 74.2–89.8) in 25–29 age group, 85.6% (95% CI: 79.6–90.3) in 30–34 age group, 89.5% (95% CI: 83.3–94.0) in 35–39 age group, and 93.3% (95% CI: 83.8–98.1) in 40–49 age group.

Overall, the anti-VZV IgG prevalence was 87.6% (95% CI: 84.4–90.3) (Figure 1), with the highest prevalence of 95.6% (95% CI: 78.0–99.9) in the 15–24 age group. Starting from the 25–29 age group, a trend towards an increase was observed among the following age groups, without any significant differences.

Considering the anti-VZV IgG prevalence for the 287 samples divided by trimester of pregnancy, the lowest proportion was found in the first trimester (80.4%, 95% CI: 71.8–87.3), although no significant differences were found among trimesters (91.8%, 95% CI: 85.8–95.8 and 87.8%, 95% CI: 73.8–95.9 in the second and the third trimester, respectively).

One sample in the 35–39 age group tested positive for both IgM and IgG, and information on trimester was not available. Another sample in the 25–29 age group in the first trimester of pregnancy tested borderline for IgM and positive for IgG.

## 4. Discussion

In this study, a high proportion of pregnant women from the province of Bari had immunity against VZV; however, 12.3% of them were susceptible to VZV infection, ranging from 4.3% to 17.0%, according to the age groups. These findings are consistent with a serological study conducted in a different province of Apulia in 2008–2009, in which 10.6% of pregnant women had no antibodies against VZV [16]. The level of susceptibility in the adult age groups did not change over time, suggesting little or no impact of the introduction of the URV. This also seems to be confirmed by comparing our results with a study conducted in Tuscany, Central Italy, in which 15.5% of women of childbearing age were found to be susceptible to VZV in 2001–2002 [18], when vaccination against VZV was not recommended.

In our study, the proportion of immune women increases with increasing age from 83.0% in the 25–29 age group to 93.3% in the 40–49 age group, consistent with other studies [16,18]. The only exception is represented by the 15–24 age group, that showed a seroprevalence to VZV of 95.6%, the highest among all age groups, likely due to the vaccination catch-up campaign targeting susceptible adolescents that was implemented in the Apulia region starting from 2009 [13]. 

While the prevalence of susceptible pregnant women in the current study is similar to other studies [19,20] and higher than in Northern European countries [16,19,21], it is lower than in other populations (i.e., in Sri Lanka) [22]. These differences in seroprevalence could be related to the country of birth, the country in which childhood is spent and educational level [19,22].

To our knowledge, this is the first study conducted in Italy to evaluate the immune status of pregnant women to VZV based on trimester of pregnancy, although we did not find a statistically significant difference between trimesters. In pregnant women with information on the gestational period, we found that a high proportion (19.6%) in the first trimester were susceptible to VZV, and therefore at a high risk of spontaneous abortion and CVS. However, a study in Norway, which tested pregnant women in the first trimesters and then at birth, showed that less than 1% seroconverted during pregnancy [21].

Notably, one sample in the first trimester of pregnancy in the 25–29 age group had a borderline result for IgM, along with another sample in the 35–39 age group that tested IgM positive, but whose pregnancy trimester was unknown. Both samples showed positivity for IgG and were likely in a seroconversion phase after VZV infection. Overall, these results show that a non-negligible proportion of pregnant women are susceptible to VZV and suggest that the virus may circulate to a wider extent than what is detected in the limited population sample included in this study. 

Moreover, newborns from susceptible mothers are not protected against VZV in the first months of life, during which varicella could be a life-threating disease [3]. 

This study had some limitations. The lack of information regarding the vaccination status or history of varicella in pregnant women involved in this study makes it difficult to interpret whether the presence of VZV antibodies is related to vaccination or to natural exposure to the wild virus. Moreover, as samples were collected in only one site, results from this study may not reflect the situation in other provinces of the Apulia region or in other Italian regions. Another limitation is the absence of any clinical information regarding the maternal/fetal outcomes in this study population.

In conclusion, our study highlights that pregnant women of 15–24 and 40–49 years of age, the youngest and the oldest, respectively, are the most protected against VZV infection, exceeding the prevalence rate of 90%. On the other hand, pregnant women between 25 and 34 years of age seem to be the most vulnerable and, considering that the mean age of first pregnancy in Italy is 31.1 years [23], the most at-risk for acquiring VZV infection during pregnancy. As for rubella and measles [15,24], analysis of immunity status against varicella should be introduced as a screening test before pregnancy, together with a strategic vaccination campaign targeting non-immune women of childbearing age, in order to reduce the risk of congenital and perinatal varicella, since the administration of a varicella vaccine during pregnancy is not recommended and pregnancy must be avoided for one month after vaccination. However, an incidental vaccination during the first month of pregnancy, or during pregnancy, is not an indicator of abortion, fetal serious complications or birth defects [25,26].

## Figures and Tables

**Table 1:** Study population by age groups; Apulia, Southern Italy, 2016–2019.

Age Groups	N	%
15–24	23	4.6
25–29	101	19.9
30–34	180	35.5
35–39	143	28.2
40–49	60	11.8
Total	507	100

**Table 2:** Study population by trimesters of pregnancy; Apulia, Southern Italy 2017–2019.

Trimester	N	%
1	112	39.0
2	134	46.7
3	41	14.3
Total	287	100
